# Convergence of Hypoxia and TGFβ Pathways on Cell Cycle Regulation in Human Hematopoietic Stem/Progenitor Cells

**DOI:** 10.1371/journal.pone.0093494

**Published:** 2014-03-31

**Authors:** Albertus T. J. Wierenga, Edo Vellenga, Jan Jacob Schuringa

**Affiliations:** 1 Department of Experimental Hematology, University Medical Center Groningen, University of Groningen, Groningen, The Netherlands; 2 Department of Laboratory Medicine, University Medical Center Groningen, University of Groningen, Groningen, The Netherlands; French Blood Institute, France

## Abstract

Although it has been shown that HIF1 and 2 fulfill essential roles within the hematopoietic system and in the regulation of HSC fate, little is currently known about the specific mechanisms that are involved. We identified transcriptome changes induced by hypoxia, constitutively active HIF1^(P402/564)^ and HIF2^(P405/531)^ in human cord blood CD34^+^ cells. Thus, we were able to identify common hypoxia-HIF1-HIF2 gene signatures, but we also identified specific target genes that were exclusively regulated by HIF1, HIF2 or hypoxia. Geneset enrichment analysis (GSEA) revealed that, besides known pathways associated with “hypoxia-induced signaling”, also significant enrichment for the Transforming Growth Factor beta (TGFβ) pathway was observed within the hypoxia/HIF1/HIF2 transcriptomes. One of the most significantly upregulated genes in both gene sets was the cyclin dependent kinase inhibitor CDKN1C (p57kip2). Combined hypoxia treatment or HIF overexpression together with TGFβ stimulation resulted in enhanced expression of CDKN1C and enhanced cell cycle arrest within the CD34^+^/CD38^−^ stem cell compartment. Interestingly, we observed that CD34^+^ cells cultured under hypoxic conditions secreted high levels of latent TGFβ, suggesting an auto- or paracrine role of TGFβ in the regulation of quiescence of these cells. However, knockdown of SMAD4 could not rescue the hypoxia induced cell cycle arrest, arguing against direct effects of hypoxia-induced secreted TGFβ. Finally, the Gα-coupled receptor GTPase RGS1 was identified as a HIF-dependent hypoxia target that dampens SDF1-induced migration and signal transduction in human CD34^+^ stem/progenitor cells.

## Introduction

Hematopoietic stem cells (HSCs) reside within specialized hypoxic niches in the bone marrow microenvironment where they are kept in a relative quiescent state [21], [24], [26], [27], [31], [34], [41]. One of the key pathways activated under low oxygen conditions is the Hypoxia-inducible factor (HIF) pathway. HIF1α and HIF2α (EPAS1) act as oxygen sensors that are degraded under normoxic conditions but at lower oxygen levels HIF proteins are stabilized, translocate to the nucleus and initiate gene transcription [20], [28], [38]. In well-oxygenated conditions HIFs are bound by the Von Hippel Lindau (VHL) tumor suppressor protein which recruits an ubiquitin ligase that targets these transcription factors for proteasomal degradation [18]. VHL binding is critically dependent on hydroxylation of proline residues in HIF1 (P405 and P564) and HIF2 (P405 and P531) [40]. The oxygen-sensitive α subunits of HIF1 or HIF2 can heterodimerize with the stable HIF1β (ARNT) subunit that together forms a basic helix-loop-helix-PAS (bHLH-PAS) transcriptional regulator that binds to the core sequence RCGTG termed the hypoxia response element (HRE) in promoters of presumed target genes [18], [20], [28], [38].

Using murine knockout models it has been shown that both HIF1α and HIF2α fulfill essential and at least in part non-overlapping roles in hematopoiesis. Conditional depletion of HIF1α resulted in loss of HSC quiescence and loss of stem cell function when exposed to stress such as transplantation, myelo-suppression or upon aging [42]. Stabilization of HIF1α, either by loss of VHL [42] or by using pharmacological inhibitors that target prolyl hydroxylases [13], resulted in increased HSC quiescence and improved hematopoietic recovery after myelosuppressive conditions. Historically, the influence of hypoxia on the behaviour of hematopoietic stem and progenitor cells has been studied in vitro by culturing murine and human bone marrow cells under reduced oxygen tension. It was shown that murine bone marrow generated roughly two-fold more CFU-GM colonies when this assay was performed under reduced (5%) oxygen conditions [2], [6]. Culturing murine or human bone marrow cells for a limited period of time under 1% oxygen conditions was shown to result in a preservation of the progenitor-generating compartment as compared to normoxic conditions [8], [17]. Furthermore, by using a transplantation model, it was shown that the repopulating activity of HSCs could be maintained or even expanded when cultured *in vitro* under reduced oxygen conditions [9], [11]. Furthermore, it was shown that long-term HSCs reside within the glycolysis-dependent subpopulation of the bone marrow that display low mitochondrial activity and express high levels of HIF1α in a Meis1-dependent manner [39]. Besides a role in HSCs, both HIF1α and HIF2α also play important role during hematopoietic development and differentiation, most notably on erythropoiesis by controlling EPO levels [15].

RGS1 is a member of the R4 subgroup of RGS proteins, known for their ability to accelerate the hydrolysis of Gα-GTP to Gα-GDP, thereby dampening the activity of GPCR signaling [5], [10]. Little is known about the specificity of the different RGS members towards different GPCR signaling, but RGS1 has been reported to be active against SDF1-induced migration of B cells by inhibiting CXCR4-mediated signaling [30]. Moreover, upregulation of RGS1 by MEIS1 and binding of MEIS1 to the promoter of RGS1 could suggest a role of RGS1 in the maintenance of HSCs [4].

Despite the critical roles of HIF1α and HIF2α in normal hematopoiesis little is known about the downstream molecular mechanisms that are involved. Therefore, we set out to identify specific and overlapping transcriptome changes induced by hypoxia, HIF1α and HIF2α in human hematopoietic CD34^+^ stem/progenitor cells. We identified that hypoxia and TGFβ pathways can converge on cell cycle regulation of HSCs by controlling genes such as CDKN1C/p57. Furthermore, we identified that RGS1 acts as a TGFβ inducible and HIF-dependent hypoxia target in human CD34^+^ stem/progenitor cells that dampens SDF1-induced migration, adhesion and signaling.

## Materials and Methods

### Cell culture and lentiviral transductions

Neonatal cord blood (CB) was obtained from healthy full-term pregnancies from the Obstetrics departments of the University Medical Center and Martini Hospital in Groningen, The Netherlands, after informed consent. The protocol was approved by the Medical Ethical Committee of the UMCG. Donors are informed about procedures and studies performed with CB by an information sheet that is read and signed by the donor, in line with regulations of the Medical Ethical Committee of the UMCG. CB CD34^+^ cells were isolated with the use of a hematopoietic progenitor isolation kit from Miltenyi Biotech according to the manufacturer’s instructions. OCI-AML3 cells (ACC-582, DSMZ, Braunschweig, Germany) were cultured in RPMI 1640 (Lonza, Verviers, Belgium) supplemented with 10% FBS, 1 mmol/L L-glutamine, and 100 U/mL penicillin/streptomycin (Life Technologies, Bleiswijk, the Netherlands). UT7/GM cells (ACC-137, DSMZ, Braunschweig, Germany) were grown in IMDM supplemented with 20% FBS, 1 mmol/L L-glutamine, 100 U/mL penicillin/streptomycin and 10 ng/ml GM-CSF.

Lentiviral vectors expressing constitutively active HIF1α(P402A,P564A) and HIF2α(P405A,P531A) were constructed by cloning the HA-tagged HIF cDNA’s, from pCDNA3 vectors obtained from Addgene (Addgene plasmid numbers 18955 and 18956) into the pRRL-SFFV-iresEGFP vector. A lentiviral vector expressing a short hairpin against SMAD4 was cloned by swapping the H1 promoter and hairpin sequence from pRetrosuper-SMAD4 (obtained from Addgene, plasmid 15727) into the pRLVPT vector [46]. pRLVPT-luc was used as the control (nonspecific) vector. A lentiviral vector expressing myc-tagged RGS1 was constructed by inserting RGS1 cDNA (obtained by RT-PCR on CB CD34^+^ derived RNA and subsequent sequencing) into the lentiviral expression vector pRRL-IRES-EGFP. Lentiviral transductions were essentially performed as described elsewhere [44]–[46]. In short, isolated CD34^+^ cells were cultured for 48 hours in serum free medium (HPGM, Lonza, Breda, the Netherlands) containing 100 ng/ml each of SCF, TPO and FLT-3 ligand. Cells were subsequently transduced with lentiviral particles in one to two consecutive rounds of 12 hours in the presence of 4 μg/ml polybrene.

### Migration assay

Migration assays were performed in transwell plates (6 micron, Costar). CB-derived CD34^+^ cells were transduced, sorted and 5×10^4^ cells per group were plated in the upper chamber. SDF1 (100 ng/ml) was added to the lower chamber and migration was allowed for 3 hours. Cells were harvested from the bottom chamber and counted by FACS using the MACSquant flow cytometer (Miltenyi Biotec).

### mRNA analysis

Total RNA was isolated using the RNeasy kit from Qiagen (Qiagen, Venlo, The Netherlands) according to the manufacturer’s recommendations. For real-time RT-PCR, cDNA was prepared using the iScript cDNA synthesis kit (Bio-Rad, Veenendaal, the Netherlands) and cycling was performed using SsoAdvanced SYBR green Supermix (Bio-Rad) in a CFX Connect thermocycler (Bio-Rad), and quantified using CFX software (Bio-Rad). HPRT and Ribosomal Protein Like (RPL) 27 expression levels were used to normalize between samples and to calculate relative expression levels. Primers and conditions are listed in table S1. Genome-wide expression analysis was performed on Illumina (Illumina, Inc., San Diego, CA) BeadChip Arrays Sentrix Human-6 (46k probesets). Typically, 0.5−1 μg of RNA combined from three to five independent transduction experiments was used in labeling reactions and hybridization with the arrays according to the manufacturer’s instructions. Data was analyzed using the BeadStudio v4 Gene Expression Module (Illumina, Inc.) and Genespring (Agilent, Amstelveen, The Netherlands). The MIAME-compliant microarray data are available at the Gene Expression Omnibus, at: http://www.ncbi.nlm.nih.gov/geo/ under accession number GSE54663.

### Flow cytometry analysis

Antibodies CD34 and CD38 were obtained from Beckton Dickinson (Breda, the Netherlands). For Hoechst and Pyronin Y staining cells were washed and resuspended in HPGM, stained in 5 μg/ml Hoechst 33342 (Invitrogen) at 37°C for 45 minutes after which 1.0 μg/ml Pyronin Y (Sigma) was added at 37°C for an additional 45 minutes. Cells were washed in the solution containing Hoechst and Pyronin Y, followed by FcR blocking at 4°C for 10 minutes. After staining with CD34-APC and CD38-Alexa700 at 4°C for 20 minutes, cells were washed and analyzed. All FACS analyses were performed on an LSRII (Becton Dickinson) or MACSquant (Milenyi Biotech) flowcytometer and data was analyzed using WinList 3D (Topsham, USA) or FlowJo (Tree Star, Oregon, USA) software.

### Immunoblotting

Sorted cells were boiled in Laemmli sample buffer for 5 min prior to separation on 10% SDS-polyacrylamide gels. Proteins were transferred to PVDF membrane (Millipore, Etten Leur, The Netherlands) by semidry electroblotting. Membranes were blocked in Odyssey blocking buffer (Westburg, Leusden, the Netherlands) prior to incubation with antibodies. Binding of antibodies was detected by incubating with Alexa680 or IRDye800 labeled secondary antibodies (Invitrogen, Breda, the Netherlands) and scanning of the membrane on an Odyssey infrared scanner (Li-Cor Biosciences, Lincoln, NE, USA). Signal intensities were quantified using Odyssey 2.1 analysis software and calculated relative to the highest intensity. Antibody against against phosporylated ERK (pT202,pY204) was obtained from Cell Signaling technologies (Leiden, the Netherlands), antibody against tyrosine phosphorylated STAT5 was obtained from Becton Dickinson (Breda, the Netherlands) and antibodies against ERK (K23) and STAT5 (C17) were obtained from Santa Cruz (Santa Cruz Biotech, Santa Cruz, CA, USA). Anti myc antibody was obtained from Roche (Almere, the Netherlands).

## Results

### Identification of hypoxia, HIF1 and HIF2 target genes in human CB CD34^+^ cells

**Figure 1:**
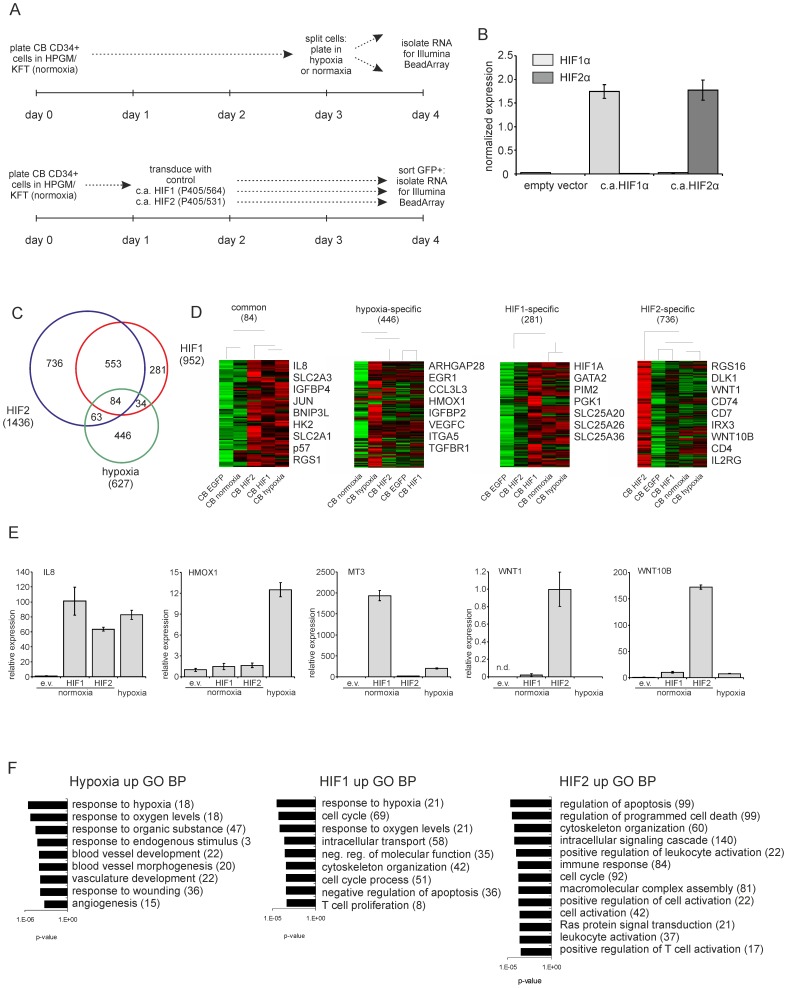
Identification of hypoxia, HIF1 and HIF2 target genes in human CB CD34^+^ cells. **A.** Schematic representation of performed experiments. **B.** Q-RT-PCR to verify HIF1/2 expression in the mRNA preparations used for transcriptome analysis. **C.** VENN diagram displays genes upregulated by HIF1, HIF2 or hypoxia. Supervised cluster analysis is shown, including a number of selected upregulated genes and associated GO terms. **D.** CB CD34^+^ cells were transduced with the indicated overexpression vectors at normoxia and/or cultured under hypoxic conditions for 24 hours. Q-RT-PCR was performed for the indicated targets. (e.v. =  empty vector, n.d. =  not detectable). **E.** Gene ontology analysis on genes upregulated by hypoxia, HIF1 or HIF2. All GO terms with an FDR<1 are shown.

In order to identify HIF1α, HIF2α and hypoxia target genes in the human hematopoietic stem/progenitor compartment, CD34^+^ cells were isolated from cord blood and were transduced with constitutively active HIF1α (P405/P564), HIF2α (P405/531) or control vectors and RNA was isolated from GFP^+^-sorted cells 24 hrs after the last transduction round (Figure 1A). Overexpression of HIF1α and HIF2α was confirmed at the RNA level (Figure 1B). Simultaneously, CB CD34^+^ cells were cultured under normoxia (21% O_2_) or hypoxia (1% O_2_) for 24 hrs followed by RNA isolation (Figure 1A). RNA from 5 separate experiments were pooled and transcriptomes were analyzed using Illumina BeadArrays. All data is summarized in Table S2. We initially focused on the HIF and hypoxia upregulated genes and significant gene expression changes in all groups are shown in the VENN diagram in Figure 1C. Thus, 84 genes could be identified that were upregulated by HIF1α, HIF2α as well as hypoxia. Furthermore, we observed a strong overlap in HIF1α and HIF2α target genes (553), although HIF1α-specific (281), HIF2α-specific (736) and hypoxia-specific (446) target genes could be identified as well. Clustering analyses were performed on these common and specific gene signatures and a number of examples of genes within these lists are indicated in Figure 1D. Q-RT-PCRs were performed to validate gene array data. A good overlap between results was obtained and some examples are shown in Figure 1E. IL8 was confirmed to be a common gene hypoxia and HIF1/2 target, HMOX1 was upregulated by hypoxia but not HIF1 or HIF2, MT3 was specifically upregulated by HIF1, and WNT1 and WNT10B were specifically upregulated by HIF2. Gene ontology (GO) analysis was performed on genes upregulated by hypoxia, HIF1 or HIF2, and all significant GO terms with an FDR<1 are shown in Figure 1F. Besides expected GO terms such as “response to hypoxia”, “response to oxygen levels”, and “angiogenesis” in hypoxia or HIF1 upregulated genes, we observed that particularly HIF2-upregulated genes were enriched for processes associated with “apoptosis” and “programmed cell death”.

**Figure 2:**
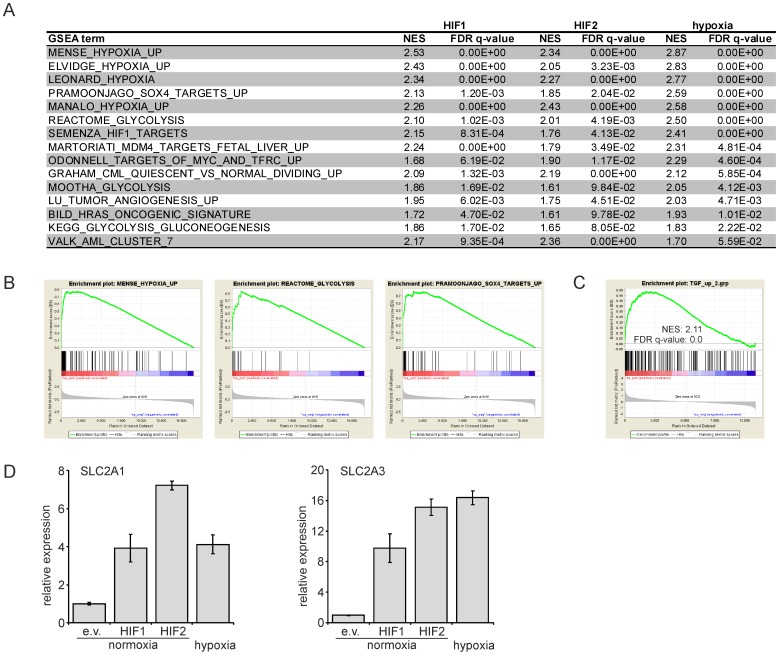
GO and GSEA analysis on hypoxia, HIF1 and HIF2 target genes. **A.** GSEA terms that are enriched in HIF1, HIF2 and hypoxia transcriptomes. **B.** Examples of enriched GSEA terms for the HIF1 transcriptomes are shown. **C.** GSEA with hypoxia transcriptome and a >2fold TGFβ upregulated dataset in CB CD34^+^ cells. **D.** CB CD34^+^ cells were transduced with the indicated overexpression vectors at normoxia and/or cultured under hypoxic conditions for 24 hours. Q-RT-PCR was performed for SLC2A1 and SLC2A3. (e.v. =  empty vector).

Next, we performed Gene set Enrichment Analysis (GSEA) on the complete HIF1, HIF2 and hypoxia transcriptomes, which revealed that Gene Ontology terms such as “hypoxia”, “glycolysis”, “angiogenesis” and “gluconeogenesis” were significantly enriched in all groups, as anticipated (Figure 2A). Somewhat more surprisingly, these analyses also revealed significant enrichment for processes associated with cellular transformation, such as “H-Ras oncogenic signature”, “SOX4 targets” and several terms associated with leukemic transformation (Figures 2A, B). Finally, we also observed significant enrichment for terms associated with TGFβ signaling. This was independently confirmed using a published dataset in which TGFβ target genes were identified in CB CD34^+^ cells [35]. Significant enrichment for the top TGFβ-upregulated genes was observed with our hypoxia (Figure 2C) and HIF1/2 (data not shown) transcriptomes.

Since we identified glycolysis was one of the processes that were enriched in our HIF and hypoxia transcriptomes we focused on expression of the glucose transporters SLC2A1 (GLUT1) and SLC2A3 (GLUT3). We compared the upregulation of these genes in our gene array study (Figure 1C) with data obtained from independent Q-PCR analyses (Figure 2D, additional Figure S1 in File S1) and observed a good correlation between both datasets, where by SLC2A1 and SLC2A3 were upregulated by hypoxia, HIF1α, as well as by HIF2α.

### Hypoxia sensitizes cells for TGFβ signaling

**Figure 3:**
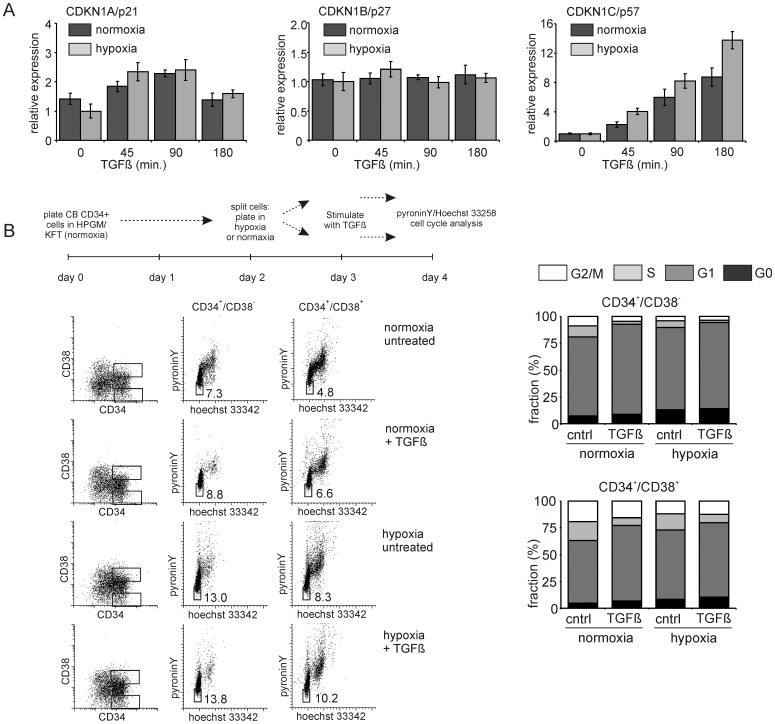
p57 and HSC quiescence are induced by both TGFβ and hypoxia. **A.** CB CD34^+^ cells were cultured under normoxic and hypoxic conditions for 24 hours and stimulated for different times with 1 ng/ml TGFβ where after Q-RT-PCR was performed for CDKN1A/p21, CDKN1B/p27 and CDKN1C/p57. A representative experiment out of 2 independent experiments is shown, error bars indicate standard deviation of PCR performed in triplicate. **B.** CB CD34^+^ cells were treated as indicated in the scheme and cell cycle analysis was performed using Hoechst and PyroninY staining**.** Representative FACS plots and the cell cycle distribution in CD34^+^/CD38^−^ and CD34^+^/CD38^+^ fractions are shown.

Since we observed a striking cooperation between TGFβ and hypoxia in activating similar target genes we decided to investigate this cooperation in further detail, in particular in relation to the cell cycle status of human hematopoietic stem/progenitor cells. We observed that the cell cycle inhibitor CDKN1C/p57 was upregulated by hypoxia, in particular when cells were co-stimulated with TGFβ (Figure 3A, Figure S2 in File S1). These effects were not observed, or at least much less pronounced on the expression of other cell cycle inhibitors such as CKN1A/p21 or CDKN1B/p27 (Figure 3A, Figure S2 in File S1). These observations could be further substantiated in studies in which we analyzed the cell cycle status and quiescence of CD34^+^/CD38^−^ stem cells and CD34^+^/CD38^+^ progenitor cells using Hoechst 33258 and Pyronin Y. Stimulation with TGFβ resulted in an increase in cells in G1 and a decrease in cells in G2/M, both in the stem compartment as well as in more committed progenitor cells (Figure 3B). Similarly, cells cultured under hypoxic conditions displayed a decrease in cells in G2/M, while an increase in quiescent cells (G0) was observed. This percentage of quiescent cells was even further increased when hypoxic cells were stimulated with TGFβ (Figure 3B).

**Figure 4:**
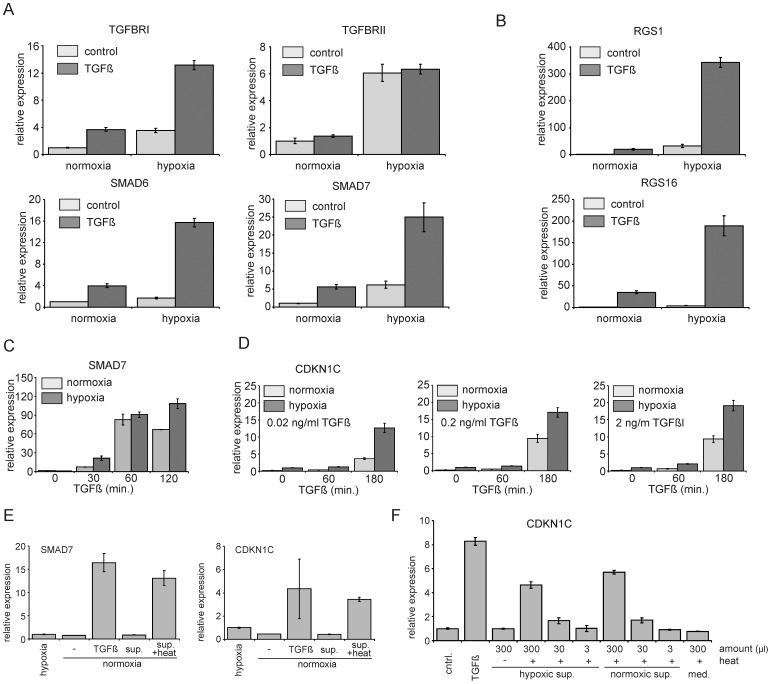
Hypoxia sensitizes cells for TGFβ signaling and CB CD34+ express latent TGFβ. **A.** OCI-AML3 cells were cultured for 24 hours under normoxic and hypoxic conditions, treated with TGFβ (1 ng/ml) for 3 hours and Q-RT-PCR was performed. **B.** OCI-AML3 cells were treated as in A, Q-RT-PCR was performed for RGS1 and RGS16. **C.** Q-RT-PCR analysis on CB CD34^+^ cells which were cultured under normoxic and hypoxic conditions and treated with 1 ng/ml TGFβ for different timepoints. **D.** CB CD34^+^ cells were treated as in C, but with different concentrations of TGFβ. **E.** CB CD34^+^ cells were cultured for 24 hours under hypoxic and normoxic conditions. Supernatant of normoxic cells was harvested and split in two, where one half was treated by incubation at 80 degrees for 10 minutes. These supernatants were added to normoxic cells for 3 hours (sup and sup + heat), along with fresh medium and fresh medium containing 1 ng/ml TGFβ. Q-RT-PCR was performed for SMAD7 and CDKN1C. **F**. Supernatant was harvested from hypoxic and normoxic cultured CB CD34^+^ cells, heat treated as in E and added back in different amounts. Heat-treated fresh medium and TGFβ containing medium was added as controls. Representative experiments out of 2−3 independent experiments is shown, error bars indicate standard deviation of PCR performed in triplicate.

Since cells appeared to be more responsive to TGFβ under hypoxic conditions we investigated whether this increased responsiveness might be explained by higher levels of TGFβ receptor expression. Quantitative RT-PCR analysis in a hypoxia and TGFβ responsive cell line (OCI-AML3) revealed that indeed both TGFBR1 as well as TGFBR2 were upregulated by hypoxia (Figure 4A, Figure S3A in File S1). In line with these observations, we noted that the well described TGFβ target genes such as SMAD6 and SMAD7 were also expressed at significantly higher levels in response to TGFβ when cells were grown under hypoxic conditions (Figure 4A, Figure S3A in File S1). These observations could be extended to hypoxia-responsive genes that had previously not been identified as classical TGFβ target genes. For instance, the Gα-coupled receptor GTPase RGS1 which we identified as one of the top genes induced by hypoxia (Figure 1D and Table S2) was even strongly further enhanced when hypoxic cells were stimulated with TGFβ (Figure 4B, Figure S3B in File S1). The same was true for the RGS R4 family member RGS16 (Figure 4B, Figure S3B in File S1). Experiments performed in cordblood CD34^+^ cells showed a similar upregulation of RGS1 and RGS16 (Figure S3D in File S1). Thus, these data strongly suggested that hypoxic conditions sensitize human stem/progenitor cells for TGFβ. To further confirm these observations, kinetics and TGFβ dosage studies were performed. CB CD34^+^ cells were cultured under normoxic or hypoxic conditions after which cells were stimulated with TGFβ for 30, 60 or 120 min. As shown in Figure 4C (and Figure S3C in File S1), exposure of cells to TGFβ for only 30 min already resulted in a strong upregulation of SMAD7, which was most prominent in cells cultured under hypoxia. Similar observations were done for CDKN1C/p57, where even at the lowest TGFβ concentrations tested, a strong upregulation of CDKN1C/p57 could be observed in cells grown under hypoxic conditions (Figure 4D).

CB CD34^+^ cells are known to express and secrete relatively high levels of latent TGFβ which still needs to be processed in order to become activated [3]. Recently, it was demonstrated that GFAP-expressing glial cells within the bone marrow are able to process latent TGFβ into its active form [47]. We questioned whether hypoxia itself, possibly via secretion and/or activation of certain proteases, might also be sufficient to activate latent TGFβ produced by human CD34^+^ cells. To address this hypothesis we undertook a series of experiments. First, we verified that human CB CD34^+^ cells indeed secrete latent TGFβ that can be activated for instance by heat treatment. Medium was harvested from cultured CB CD34^+^ cells both under normoxic as well as hypoxic conditions, was heat treated at 80°C and then used to stimulate human CB CD34^+^ cells. As shown in Figure 4E, this heat-treated medium was equally capable of inducing SMAD7 or CDKN1C/p57 expression as compared to treatment with TGFβ. Furthermore, this induction of SMAD7 and CDKN1C/p57 could be inhibited by either treatment with antagonizing anti-TGBβ antibodies or by downmodulation of SMAD4 using lentiviral shRNA vectors (data not shown), indicating the specificity of the responses induced by the heat-treated conditioned medium. Conditioned medium that was harvested from cells grown under hypoxic conditions was not able to induce the TGFβ target genes CDKN1c/p57 (Figure 4F) and SMAD7 (not shown), and no differences were observed when different amounts of heat-treated conditioned medium was used from cells frown under normoxic or hypoxic conditions. These data strongly suggest that hypoxia itself is not sufficient to activate latent TGFβ.

### TGFβ, but not hypoxia, induced cell cycle arrest is SMAD dependent

**Figure 5:**
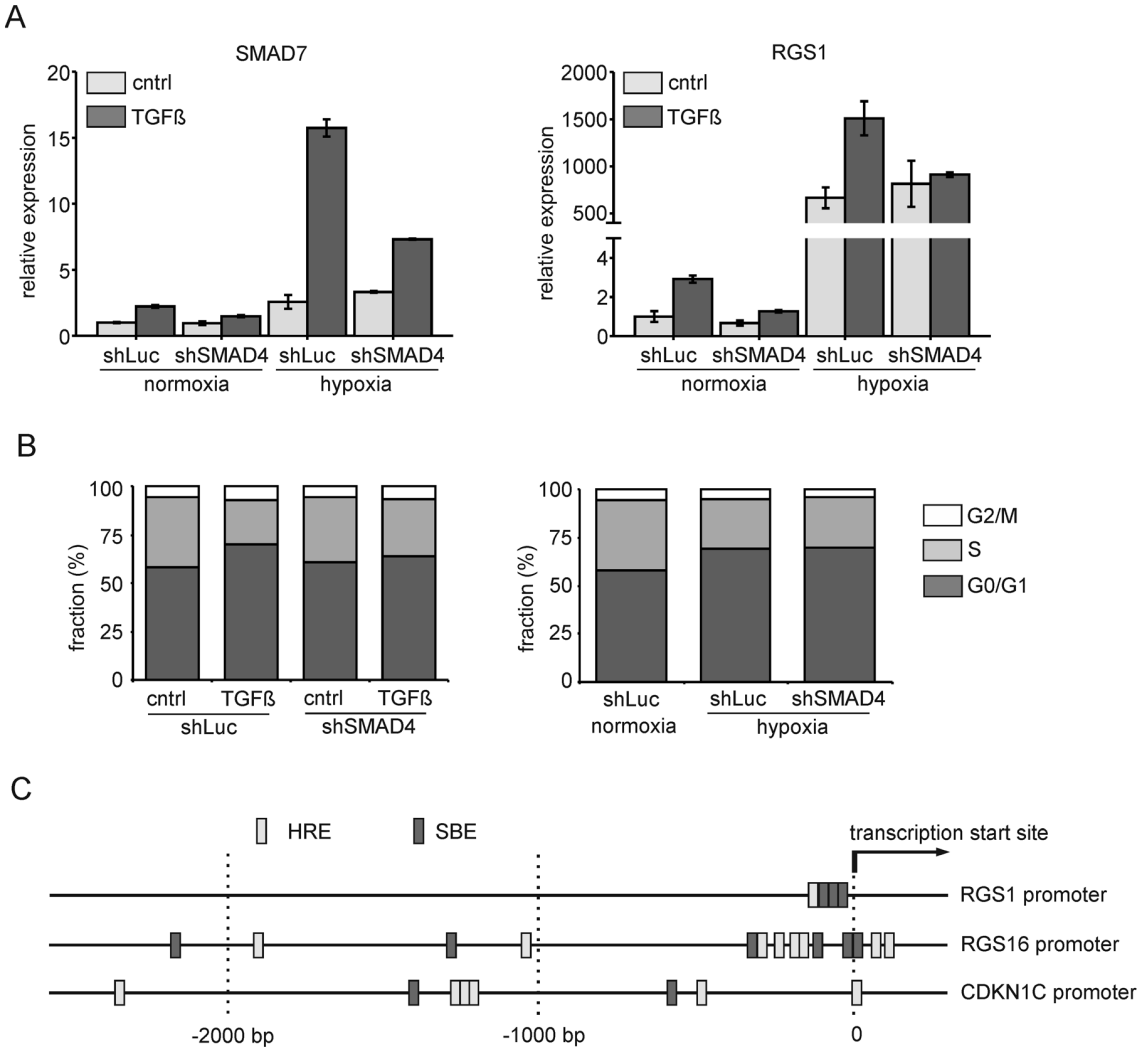
TGFβ, but not hypoxia, induced cell cycle arrest is SMAD4 dependent. **A.** CB CD34^+^ cells were transduced with vectors expressing short hairpins against SMAD4 and luciferase, sorted and cultured for 24 hours under normoxic and hypoxic conditions. Cells were then stimulated for 3 hours with 1 ng/ml TGFβ and Q-RT-PCR was performed for SMAD7 and RGS1. A representative experiment out of 3 independent experiments is shown, error bars indicate standard deviation of PCR performed in triplicate. **B.** CB CD34^+^ cells were transduced as in A but now cultured for 24 hours under normoxia (left panel) and normoxia versus hypoxia (right panel) and subsequently treated with TGFβ for 24 hours. Cell cycle distribution was measured using Hoechst staining. **C.** Schematic representation of the 2,5 kb upstream promoter region of RGS1, RGS16 and CDKN1C.

Subsequently we investigated whether hypoxia-induced expression of target genes and cell cycle arrest would be SMAD-signaling dependent. Using OCI-AML3 cells, SMAD4 was downregulated using shRNA lentiviral vectors, which efficiently inhibited TGFβ induced expression of SMAD7 or RGS1, both under normoxic as well as hypoxic conditions (Figure 5A). However, hypoxia-induced expression of SMAD7 and RGS1 did not depend on SMAD4 (Figure 5A). Similarly, we observed that TGFβ-induced cell cycle arrest in CB CD34^+^ cells, but not hypoxia-induced cell cycle arrest, depended on SMAD4 (Figure 5B). These data indicate that TGFβ and hypoxia pathways do not act synergistically but rather operate side by side to activate similar target genes. Indeed, we observed that CDKN1C/P57, RGS1 and RGS16 promoters all contain potential binding sites for SMAD complexes (SBEs) as well as HIF transcription factors (HREs) (Figure 5C).

### RGS1 dampens SDF1 induced migration, adhesion and signaling in CB CD34^+^ cells

**Figure 6:**
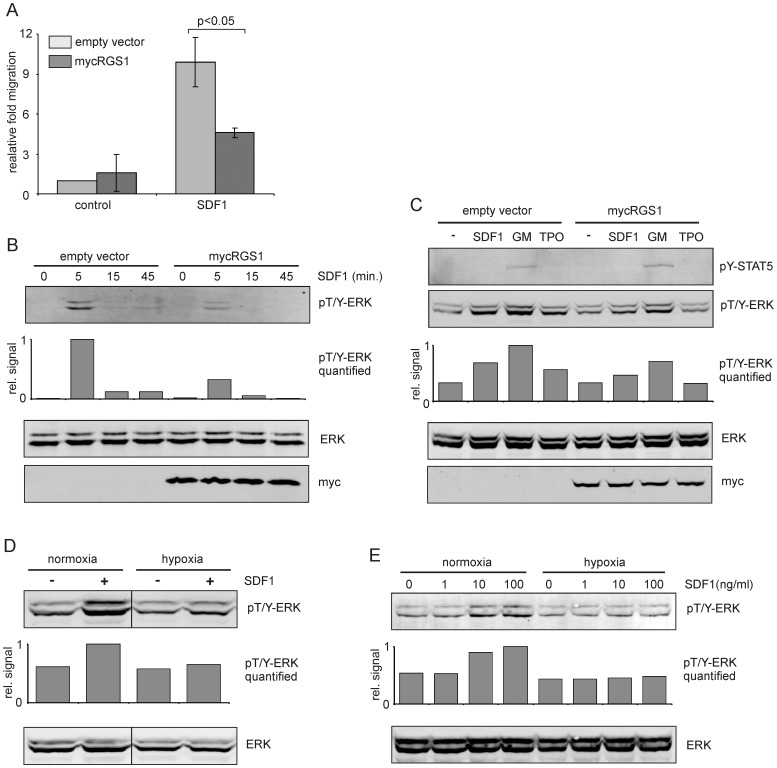
RGS1 dampens SDF1-induced migration and signaling. **A.** Transwell migration assay towards SDF1 (100 ng/ml) using CB CD34^+^ cells transduced with empty vectors or RGS1. The mean and standard deviation of three independent experiments is shown. **B.** Western blot of CD34^+^ cells transduced with empty vectors or RGS1 that were stimulated with SDF1 (20 ng/ml) for different timepoints. **C.** Western blot of OCI-AML3 cells transduced with empty vectors or RGS1 that were stimulated with different cytokines (20 ng/ml each) for 10 minutes. **D.** OCI-AML3 cells were cultured for 24 hours under normoxia or hypoxia, stimulated with 20 ng/ml SDF1 for 10 minutes and Western blotted for phosphorylated ERK1/2. **E.** UT7-GM cells were cultured for 24 hours under normoxia or hypoxia, stimulated with 20 ng/ml SDF1 for different timepoints and Western blotted for phosphorylated ERK1/2. Band intensities for phospho-ERK1/2 were measured and plotted as relative signal (in B-E). Representative experiments out of 2-3 independent experiments are shown.

RGS1 is a member of the R4 subgroup of RGS proteins, known for their ability to accelerate the hydrolysis of Gα-GTP to Gα-GDP, thereby dampening the activity of GPCR signaling [10]. To test the hypothesis that RGS1, induced by hypoxia and/or TGFβ, could influence the strength of specific signaling cascades, cells were lentivirally transduced with myc-tagged RGS1 and the response to SDF-1 stimulation was determined by migration assays as well as Western blots. RGS1 overexpression clearly resulted in a strongly (approximately 50%) reduced migration of CB CD34^+^ cells towards 100 ng/ml SDF1 (Figure 6A). The ERK/MAPK pathway is one of the signaling cascades that can be induced by SDF-1, and overexpression of RGS1 resulted in a significant reduction in SDF-1-induced ERK phosphorylation in CB CD34^+^ cells (Figure 6B). In line with these results, stimulation of OCI-AML3 cells with SDF1 but also with GM-CSF or TPO revealed that ERK phosphorylation is dampened by overexpression of RGS1 (Figure 6C, Figure S4 in File S1). In contrast, GM-CSF-induced STAT5 phosphorylation was not affected by RGS1 overexpression (Figure 6C, Figure S4 in File S1). Culturing OCI-AML3 cells or UT7-GM cells under hypoxic conditions for 24 hours before stimulation with SDF1 revealed that also under these conditions ERK phosphorylation was reduced, suggesting an important role for RGS1 in controlling cytokine signaling under hypoxic conditions (Figure 6D and E).

## Discussion

Despite a high degree of sequence homology between HIF1 and HIF2, specific functions have been described for each [38]. HIF1^−/−^ embryos suffer from severe cardiac and vascular malformations and die by E10.5 [23]. HIF2^−/−^ mice also display vascular defects and die by E12.5 [32] or a couple of months after birth due to ROS-mediated multiorgan failure and metabolic abnormalities [37], depending on the genetic background of the mouse strain used. Conditional knockout models have revealed that HIF1 is required to maintain HSC quiescence and loss of HIF1 impairs stem cell function when exposed to stress such as serial transplantation, myelo-suppression or upon aging [42]. In contrast, constitutive or inducible loss of HIF2 did not affect steady state hematopoiesis, HSC numbers or serial repopulating capacity of HSCs [14], although a recent paper in human CB CD34^+^ cells indicated that knockdown of HIF2α did impede long-term repopulating activity [33]. Our data indicate that within human hematopoietic CD34^+^ stem/progenitor cells HIF1 and HIF2 induce overlapping but also distinct gene expression patterns. Glucose metabolism genes such as HK2, SLC2A1 and SLC2A3 can be activated by both HIF1 and HIF2, and the same is true for IL8, CXCR4 and CDKN1C. The zinc-binding protein Metallothionein 3 (MT3) is very specifically upregulated by HIF1 in human CD34^+^ cells, although little is known about its role in hematopoiesis. HIF2-specific target genes include WNT1 and WNT10B. Gene ontology analysis revealed that a subset of genes associated with apoptosis is also preferentially controlled by HIF2. It is important to realize that changes in RNA expression might not always directly reflect changes at the protein level, particularly under low oxygen conditions [12]. Nevertheless, our data do clearly indicate that besides common HIF regulated genes, HIF1 and HIF2 specific genes exist as well. These data are in line with recent ChIP-seq experiments in MCF-7 breast cancer cells in which genome-wide HIF1 and HIF2 binding sites were mapped [36]. We find a good overlap with glucose metabolism and oxidative stress related genes when comparing their datasets with ours although differences exist as well, and for instance no direct binding was observed on IL8, CXCR4 or MT3 loci by Schödel et al [36]. Thus, these targets might indirectly be controlled by HIF1 or 2, or it is also plausible that differences in HIF targets between cell types might exist.

We have also been able to identify genes that are upregulated by hypoxia, but not by HIF1 or HIF2. These include for instance ARHGAP28, KLF2, EGR1 and HMOX1. HMOX1 expression has been shown to be regulated by Nuclear factor erythroid 2-Related factor 2 (NRF2) [1]. NRF2 is a global regulator of the oxidative stress response, but has recently also been shown to regulate several aspects of hematopoietic stem cell homeostasis [43]. Upon exposure to oxidative stress NRF2 is released from its negative regulator KEAP and thereby escapes from degradation, allowing nuclear translocation and activation of target genes [22]. A recent study indicated that hypoxia-induced HMOX1 expression is repressed upon downmodulation of NRF2 [25], strongly suggesting that this pathway, but not the HIF pathway, controls HMOX1 expression under hypoxic conditions. The transcription factor EGR1 is highly expressed in the most primitive hematopoietic stem cell compartment where it critically regulates self-renewal as well the localization of HSCs within the bone marrow niche [29]. Loss of one allele of HMOX1 is sufficient to impair radioprotection and serial repopulation of HSCs [7]. Together, these results indicate that, besides the HIF pathway, other hypoxia-sensitive signaling routes including the NRF2 pathway fulfill essential roles in HSCs within the hypoxic bone marrow niche.

Besides hypoxia and glucose metabolism signatures, gene set enrichment analysis revealed a remarkable overlap of HIF/hypoxia targets with genes controlled by TGFβ. A key example of one such gene is the cell cycle inhibitor p57/CDKN1C. While the expression of other cell cycle regulators such as p21/CDKN1A or p27/CDKN1B was hardly affected by either hypoxia or TGFβ treatment, the expression of p57/CDKN1C was strongly induced by TGFβ in a dose-dependent manner, in particular under hypoxic conditions. In part this may be explained by an increase in TGFBR1 and TGFBR2 expression in upon hypoxia. These data suggest that cells cultured under hypoxic conditions become hypersensitive to TGFβ, a feature that was also observed for other genes such as RGS1 and RGS16 that were most strongly upregulated by TGFβ under hypoxia. These data are of particular interest since it was demonstrated that TGFβ induces HSC hibernation [48]. We also confirmed that TGFβ can induce a cell cycle arrest and increases quiescence of HSCs and MPPs, in particular under hypoxic conditions. In vivo this might be mediated by non-myelinating Schwann cells within the bone marrow niche that provides factors that can activate latent TGFβ which was essential for HSC maintenance [47]. Deletion of SMAD4, a critical downstream mediator of canonical TGFβ signaling impaired HSC self-renewal and repopulation capacity [19]. Clearly, TGFβ signaling is a critical mediator of hematopoietic stem cell fate, in part by controlling the expression of p57/CDKN1C [35] and it is intriguing that HSCs are particularly sensitive to TGFβ stimulation under hypoxic conditions which are present in the bone marrow niche.

Human CD34^+^ cells themselves express high levels of latent TGFβ, and we hypothesized that this might be activated by hypoxia itself? possibly by inducing the activation and/or secretion of certain proteases within CD34^+^ cells. However, we have not found evidence for this hypothesis, and it appears more likely that other niche components, such as for instance non-myelinating Schwann cells, are required to activate TGFβ within the hypoxic bone marrow microenvironment.

RGS1 was identified as a common gene that is upregulated by hypoxia, HIF1 and HIF2. Although little is known about its role in hematopoietic stem/progenitor cells, it has been shown that RGS1 can reduce migration of B cells towards SDF1 [16] and can be upregulated by IFNb-1β in monocytes, T cells and B cells. RGS proteins interfere with G-protein signaling by accelerating the hydrolysis of Gα-GTP to Gα-GDP, thereby dampening the activity of GPCR signaling [10]. Indeed, we observed that migration of human CB CD34^+^ cells towards SDF1 was impaired by RGS1 overexpression or when cells were grown under hypoxia, coinciding with a reduced phosphorylation of ERK. Besides SDF1 signaling, activation of the MAPK/ERK pathway by GM-CSF or TPO was also impaired by RGS1 overexpression, while GM-CSF-induced activation of STAT5 was unaltered. These data suggest that RGS1 exerts its effects on a G-protein coupled receptor upstream of the MAPK/ERK pathway, thereby possibly affecting RAS activity. Our data indicate that when cells encounter hypoxic conditions they respond differently to cytokines and growth factors and it will be interesting to further elucidate the underlying mechanisms and consequences thereof for the fate of hematopoietic stem and progenitor cells.

## Conclusion

In conclusion, our work reveals common hypoxia-HIF1-HIF2 gene signatures in human CD34^+^ stem/progenitor cells, but we also identified specific target genes that are exclusively regulated by HIF1, HIF2 or hypoxia. A strong overlap between these common gene signatures and TGFβ-regulated genes exists and a key example of one such gene is the cell cycle regulator p57/CDKN1C. Combined hypoxia treatment or HIF overexpression together with TGFβ stimulation resulted in enhanced expression of CDKN1C and enhanced cell cycle arrest within the CD34^+^/CD38^−^ stem cell compartment. Finally, the Gα-coupled receptor GTPase RGS1 was identified as a HIF-dependent hypoxia target that dampens SDF1-induced migration and signaling in human CD34^+^ stem/progenitor cells.

## Supporting Information

File S1 **Supplemental data for the manuscript.** Figure S1, Figure S2, Figure S3, Figure S4.
(PDF)

Table S1 (DOC)

Table S2 **Transcriptome data.**
(XLS)
