# Supplementary material for: Convergence of Hypoxia and TGFβ Pathways on Cell Cycle Regulation in Human Hematopoietic Stem/Progenitor Cells
Source: PLoS One. 2014 Mar 31;9(3):e93494. doi: 10.1371/journal.pone.0093494 (PMC3970968; doi:10.1371/journal.pone.0093494)
Supplement: File S1 — Supplemental data for the manuscript. Figure S1, Figure S2, Figure S3, Figure S4. (PDF) [file pone.0093494.s001.pdf]

Supplemental File S1:
Supplemental data for the manuscript

Title: Convergence of hypoxia and TGFβ pathways on cell cycle regulation in human hematopoietic stem/progenitor cells

Albertus T.J. Wierenga[1,2], Edo Vellenga[1] and Jan Jacob Schuringa[1].

[1]Department of Experimental Hematology, [2]Department of Laboratory Medicine, University Medical Center Groningen, University of Groningen, Hanzeplein 1, 9700 RB, Groningen, The Netherlands.

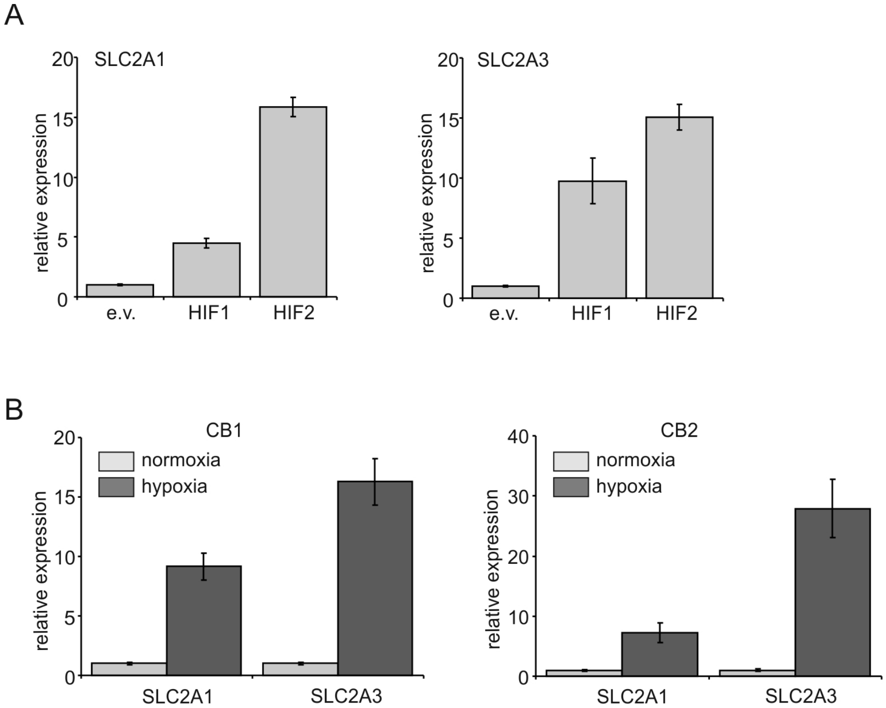

Figure S1. Verification of HIF1, HIF2 and hypoxia target genes. A. CB CD34$^+$ cells were transduced with the indicated overexpression vectors at normoxia. Cells were harvested 24 hours after transduction and Q-RT-PCR was performed for SLC2A1 and SLC2A3. (e.v.= empty vector). **B**. CB CD34$^+$ cells were cultured for 24 hours at normoxia and hypoxia, after which Q-RT-PCR was performed for SLC2A1 and SLC2A3. Two independent cord blood batches are shown. Error bars indicate standard deviation of PCR performed in triplicate.

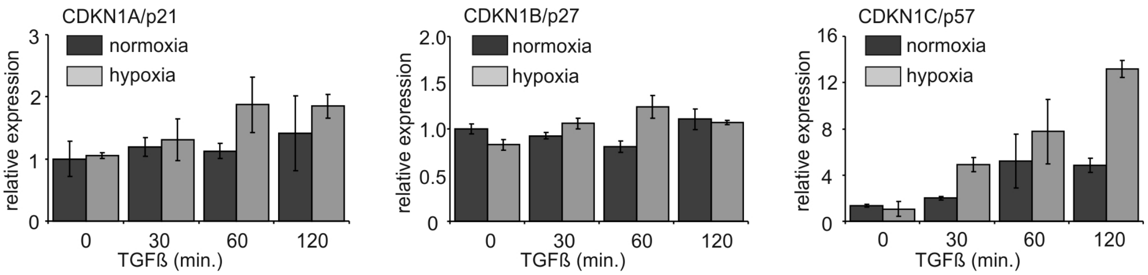

Figure S2. CDKN1A, CDKN1B and CDKN1C gene induction by TGFβ and hypoxia. CB $CD34^{+}$ cells were cultured under normoxic and hypoxic conditions for 24 hours and stimulated for different times with 1 ng/ml TGFβ where after Q-RT-PCR was performed for CDKN1A/p21, CDKN1B/p27 and CDKN1C/p57. Error bars indicate standard deviation of PCR performed in triplicate.

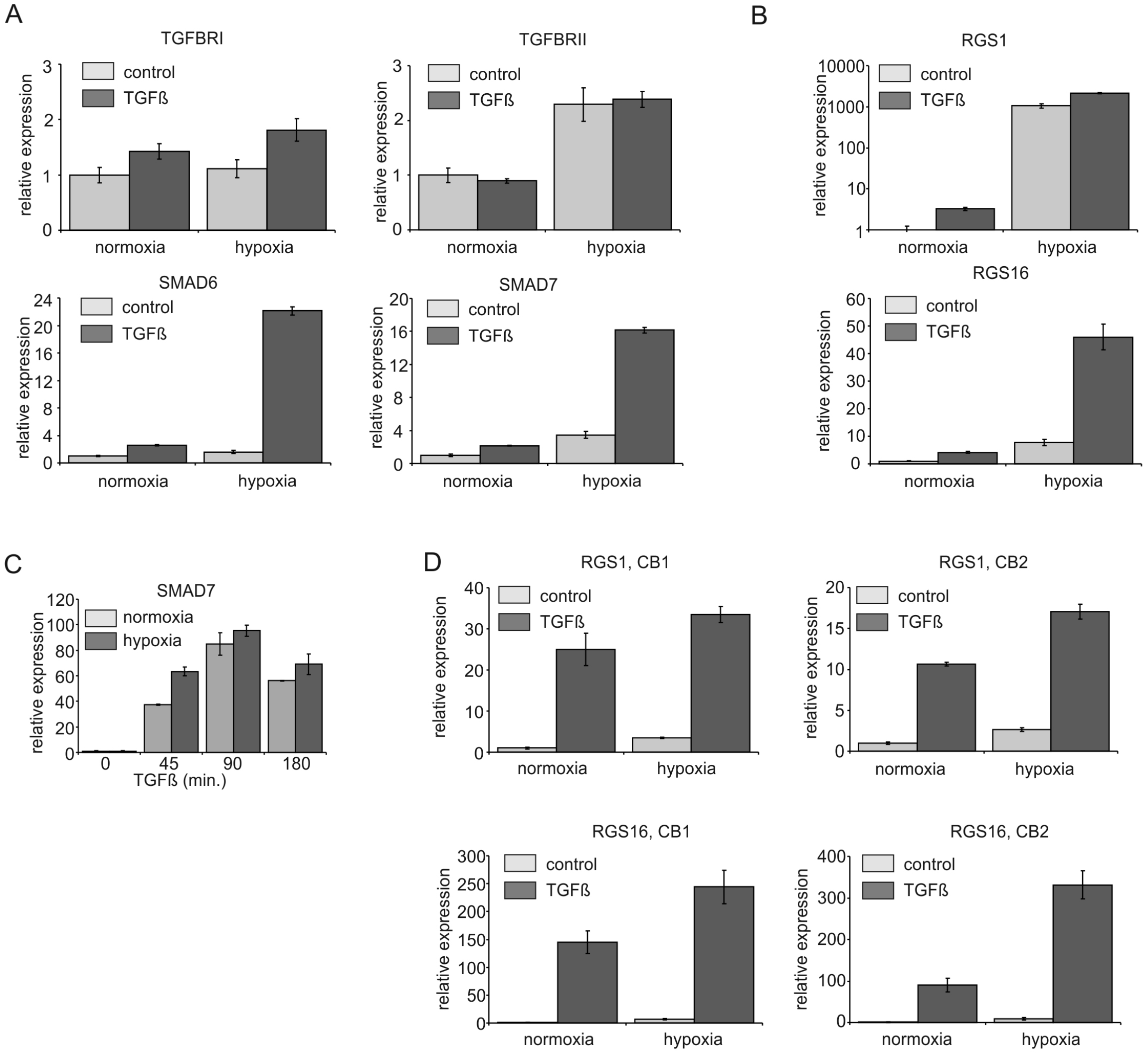

Figure S3. Hypoxia sensitizes cells for TGFβ signaling.

A. OCI-AML3 cells were cultured for 24 hours under normoxic and hypoxic conditions, treated with TGFβ (1 ng/ml) for 3 hours and Q-RT-PCR was performed. **B**. OCI-AML3 cells were treated as in A, Q-RT-PCR was performed for RGS1 and RGS16. **C**. Q-RT-PCR analysis on CB CD34$^+$ cells which were cultured under normoxic and hypoxic conditions and treated with 1 ng/ml TGFβ for different timepoints. **D**. Same experiments as in B with CB CD34$^+$ cells. Two independent cord blood batches were used (CB1 and CB2). Error bars indicate the standard deviation of the PCR performed in triplicate.

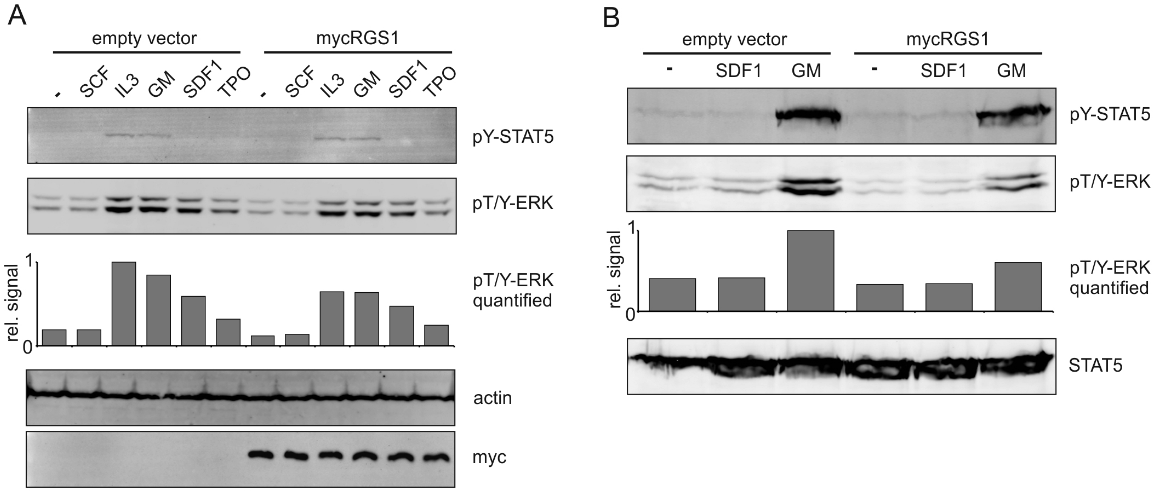

Figure S4. Reduced ERK signaling upon overexpression of RGS1

A. Western blot of OCI-AML3 cells transduced with empty vector or vector expressing mycRGS1 that were stimulated with different cytokines (20 ng/ml each) for 10 minutes. Equal loading was confirmed by blotting against actin. **B.** UT7-GM cells, overnight deprived from GM-CSF, were stimulated with SDF1 or GM-CSF (20 ng/ml, 10 minutes). Western blot was performed using the indicated antibodies. Total STAT5 probing shows equal gel loading. Band intensities for phospho-ERK1/2 were measured and plotted as relative signal.
