# Supplementary material for: Convergence of Hypoxia and TGFβ Pathways on Cell Cycle Regulation in Human Hematopoietic Stem/Progenitor Cells
Source: PLoS One. 2014 Mar 31;9(3):e93494. doi: 10.1371/journal.pone.0093494 (PMC3970968; doi:10.1371/journal.pone.0093494)
Supplement: Table S1 — (DOC) [file pone.0093494.s002.doc]

**Supplemental table S1, primer sequences**

|  | forward | reverse |
| --- | --- | --- |
| HIF1A | agttccgcaagccctgaaag | gtggtagtggtggcattagc |
| HIF2A/EPAS1 | ctgcgaccatgaggagattc | tgacagtacggcctctgttg |
| SMAD6 | tactctcggctgtctcctc | gagttggtagcctccgtttc |
| SMAD7 | agatgctgtgccttcctc | ccaggctccagaagaagttg |
| TGFBR1 | accttctgacccatcagttg | tagctgctccattggcatac |
| TGFBR2 | gagaagccacaggaagtc | gggtcatggcaaactgtc |
| RGS1 | tgccctgtaaagcagaag | tggctgtagattctcgagtg |
| RGS16 | gtactggcaagttcgagtg | cttcaggaaagcgtggaag |
| WNT1 | cttcggcaagatcgtcaacc | cgtgcaggattcgatggaac |
| WNT10B | aggcacgaatgcgaatcc | ccctccagcatgtcttgaac |
| MT3 | ttgcttggagaagcccgttca | gcatttgcatccctcgcacttg |
| CDKN1A | actaggcggttgaatgagag | aggaagtagctggcatgaag |
| CDKN1B | cgacctgcaaccgacgattc | gtcttctgaggccaggcttc |
| CDKN1C | aagagatcagcgcctgagaag | tgggctctaaattggctcacc |
| IL8 | cagccttcctgatttctg | gggtggaaaggtttggagtatg |
| SLC2A1 | ttgtgggcatgtgcttccag | atcgaaggtccggcctttag |
| SLC2A3 | cgtcggactcttcgtcaac | caccagtgacagccaacag |
| HMOX1 | gcctggccttcttcaccttc | gtcagcagctcctgcaactc |

**PCR conditions:**

**10’ 95ºC**

**15’ 58ºC**

**40 cycles**
